# Sanitary Suggestions on How to Disinfect Our Homes

**Published:** 1885-07

**Authors:** 


**Sanitary Suggestions on How to Disinfect our Homes.** A resume of the latest and best information on the household use of disinfectants, deodorants and antiseptics, and of practical precautions preventive of cholera, diphtheria, scarlet fever and other infectious diseases. B. W. Palmer, M. D. Paper. Price, 25 cents. Geo. S. Davis, publisher, Detroit, Mich.

A very useful little book. It contains much valuable instruction in a form adapted for popular perusal. Its cost is trifling, and physicians may well commend it to their patients and save themselves the necessity of repeated explanations and directions as to the necessity and use of disinfectants.

